# Transforming Growth Factor β Neutralization Ameliorates Pre-Existing Hepatic Fibrosis and Reduces Cholangiocarcinoma in Thioacetamide-Treated Rats

**DOI:** 10.1371/journal.pone.0054499

**Published:** 2013-01-17

**Authors:** Hong Ling, Eric Roux, Donna Hempel, Jingzang Tao, Mandy Smith, Scott Lonning, Anna Zuk, Cynthia Arbeeny, Steve Ledbetter

**Affiliations:** Tissue Protection and Repair, Sanofi-Genzyme R&D Center, Framingham, Massachusetts, United States of America; Medical University Graz, Austria

## Abstract

Considerable evidence has demonstrated that transforming growth factor β (TGF-β) plays a key role in hepatic fibrosis, the final common pathway for a variety of chronic liver diseases leading to liver insufficiency. Although a few studies have reported that blocking TGF-β with soluble receptors or siRNA can prevent the progression of hepatic fibrosis, as yet no evidence has been provided that TGF-β antagonism can improve pre-existing hepatic fibrosis. The aim of this study was to examine the effects of a murine neutralizing TGF-β monoclonal antibody (1D11), in a rat model of thioacetamide (TAA)-induced hepatic fibrosis. TAA administration for 8 weeks induced extensive hepatic fibrosis, whereupon 1D11 dosing was initiated and maintained for 8 additional weeks. Comparing the extent of fibrosis at two time points, pre- and post-1D11 dosing, we observed a profound regression of tissue injury and fibrosis upon treatment, as reflected by a reduction of collagen deposition to a level significantly less than that observed before 1D11 dosing. Hepatic TGF-β1 mRNA, tissue hydroxyproline, and plasminogen activator inhibitor 1 (PAI-1) levels were significantly elevated at the end of the 8 week TAA treatment. Vehicle and antibody control groups demonstrated progressive injury through 16 weeks, whereas those animals treated for 8 weeks with 1D11 showed striking improvement in histologic and molecular endpoints. During the course of tissue injury, TAA also induced cholangiocarcinomas. At the end of study, the number and area of cholangiocarcinomas were significantly diminished in rats receiving 1D11 as compared to control groups, presumably by the marked reduction of supporting fibrosis/stroma. The present study demonstrates that 1D11 can reverse pre-existing hepatic fibrosis induced by extended dosing of TAA. The regression of fibrosis was accompanied by a marked reduction in concomitantly developed cholangiocarcinomas. These data provide evidence that therapeutic dosing of a TGF-β antagonist can diminish and potentially reverse hepatic fibrosis and also reduce the number and size of attendant cholangiocarcinomas.

## Introduction

Liver cirrhosis is a common end consequence of a variety of chronic liver diseases. Its underlying pathology, fibrosis, represents the common response of the liver to toxic, infectious, or metabolic agents [1]–[3]. Hepatic fibrosis, i.e., excess deposition of extracellular matrix proteins, is traditionally viewed as an irreversible pathological process involving multiple cellular and molecular events [2], [4]–[5]. In most patients with liver cirrhosis, disease pathology increases in severity and does not regress, leading ultimately to liver insufficiency and to the development of liver carcinoma. However, recent evidence suggests that liver fibrosis is dynamic and can be bidirectional, involving phases of progression and regression [6], offering an opportunity for therapeutic intervention to halt or reverse progression.

Transforming growth factor β (TGF-β) is a pleiotropic cytokine, which regulates numerous essential cell functions. Considerable evidence has accumulated showing that excess expression of TGF-β induces and orchestrates intracellular signaling events leading to increased matrix protein deposition and ultimately liver fibrosis [7]–[9]. TGF-β1 is the main isoform mediating liver fibrosis through autocrine and paracrine effects on various hepatic and infiltrating cell types [7]–[9]. This pathological process also involves major changes in the regulation of matrix degradation, in which plasminogen activator inhibitor 1 (PAI-1), a downstream effector of TGF-β signaling, may be a key player [10]–[11]. TGF-β mediated changes to the structure and biophysical properties of the extracellular micro-environment may also promote the appearance and growth of neoplastic epithelial cells (16). However, the role of TGF-β in this context is complex as this molecule also promotes epithelial mesenchymal transdifferentiation (EMT), cell invasiveness and metastasis [12]–[13], whereas in other settings TGF-β functions as a tumor suppressor [14]–[15].

Given the prominent role of TGF-β in hepatic fibrosis, several approaches to abrogate the effect of TGF-β have been reported. These therapeutic strategies have been shown to be effective in preventing liver fibrosis in several animal models. For example, adenovirus-mediated local expression of dominant negative type II TGF-β receptor (TβRII) in liver and skeletal muscle significantly reduced the extent of hepatic fibrosis in a thioacetamide (TAA)-induced liver fibrosis model [16]. Additionally, engineered forms of soluble TGF-β receptor II, which act as a scavenger of this cytokine, or RNA interference targeting TGF-β1, prevent fibrogenesis in rodent models of liver disease [17]–[19]. These studies have clearly established an anti-fibrotic role for TGF-β antagonists in preventing liver fibrogenesis. However, the agents were administered at the time of injury, at an early stage of disease when substantial fibrosis was not yet developed, or in models that could spontaneously regress after the toxic agents were removed. Therefore, these studies do not address the therapeutic utility of TGF-β antagonism in a setting of pre-existing hepatic fibrosis. The aim of the present study was to investigate the effects of a TGF-β neutralizing antibody, 1D11, in a rat model of TAA-induced hepatic fibrosis, accompanied with the development of cholangiocarcinoma (CCA) that recapitulates the histological features and progression of human CCA [20]–[21]. The results suggest that antagonizing TGF-β may reverse pre-existing hepatic fibrosis by disrupting TGF-β synthesis, reducing extracellular matrix production and promoting matrix degradation. Unexpectedly, this therapeutic approach also substantially reduced TAA-induced CCA.

## Materials and Methods

### Ethics Statement

This study was carried out in strict accordance with the recommendations in the Guide for the Care and Use of Laboratory Animals of the National Institutes of Health. All protocols were approved by Genzyme’s Institutional Animal Care and Use Committee (permit Number: 03-0918-2-BC).

### Antibody Preparation

A murine IgG1 monoclonal antibody, 1D11, which neutralizes all three mammalian TGF-β isoforms (β1, β2 and β3), was produced at Genzyme Corporation (Framingham, MA). This antibody has a circulatory half-life of 5.5 days in rats when administered by intraperitoneal injection. An isotype-matched irrelevant murine IgG1 monoclonal antibody, 13C4, also produced by Genzyme Corporation, was used as a control antibody.

### Experimental Protocol

**Figure 1:**
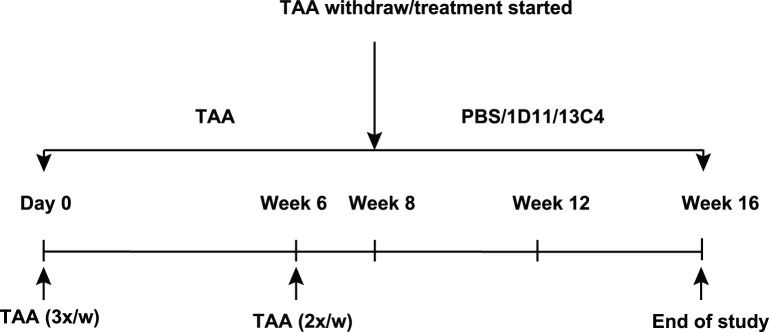
Experimental protocol of TAA induced liver fibrosis. Fischer rats were dosed with TAA intraperitoneally for 8 weeks (300 mg/kg for 6 weeks, three times weekly, then two times weekly for 2 weeks). A cohort of rats received PBS as normal controls. At the end of 8 weeks, TAA was withdrawn and rats were divided into three cohorts that were given PBS, 13C4 or 1D11 for 8 weeks. 13C4 and 1D11 were dosed at a concentration of 5 mg/kg, intraperitoneally, three times per week.

Adult Fischer 344 rats (Charles River Laboratories, Worchester, MA) weighing 280 grams (8–10 weeks old) were housed in an air-, temperature-, and light-controlled environment. Based on our pilot studies, the TAA-induced hepatic fibrosis model in Fischer rat (F344) was chosen as this model has irreversible hepatic fibrosis which is consistent among individual animals, and disease pathology is comparable to humans. Experimental design and dosing regimen are shown in Figure 1. For these studies, rats were dosed with TAA intraperitoneally for 8 weeks (300 mg/kg three times weekly for 6 weeks, followed by two times weekly for 2 weeks, Figure 1). A group of rats received vehicle buffer (phosphate buffered saline, PBS) and served as normal controls. At the end of 8 weeks, TAA was withdrawn and six rats were sacrificed to establish pre-existing hepatic fibrosis. The remaining animals were divided into three groups and were given PBS, 13C4 or 1D11. 13C4 and 1D11 were dosed at 5 mg/kg, 3 times per week, IP. At sacrifice, livers were perfused with sterile PBS. Sample collection included harvesting the identical portion of the right lobe of the liver to avoid lobe to lobe variability. Samples were then quickly frozen on dry ice and stored at −80°C until use. Samples were processed for the analysis of mRNA, measurement of hydroxyproline content, or Western blotting. Freshly harvested liver samples were also fixed in 10% buffer neutralized formaldehyde for 24 hours for histopathological examination, morphometric analysis and immunostaining.

### Blood Chemistry Analysis

Blood was drawn at week 0, 3 and 8 (with TAA dosing), and 11 and 14 weeks (3 and 6 weeks post treatments, respectively). Plasma samples were analyzed for albumin and liver enzymes, alanine aminotransferase (ALT), and aspartate aminotransferase (AST) using a Roche Cobas Automate Biochemistry Analyzer (Roche Diagnostics, Indianapolis, IN).

### Histopathological Assessment

Paraffin embedded liver sections (5-µm) were stained with hematoxylin-eosin (H&E) or picrosirius red. Assessment of the histopathological changes was conducted by applying conventional semi-quantitative scoring system for fibrosis, as described [22]. All evaluations were conducted blindly. Briefly, histopathological changes were assessed based on biliary epithelial neoplasia, fibrous deposition, and fibrous bridging between portal areas or between portal areas and central veins, with a greater attention to the extent of collagen deposition. Semi-scores were given by estimating the lesions per section and the completion of fibrous bridging, as follows: 0 = none, 1 = <25%, 2 = 25–50%, 3 = <75% (with full bridging, cirrhotic), and 4 = >75% (severe cirrhotic), respectively. Data are expressed as mean ± SE.

### Morphometric Analysis

Morphometric analysis was performed on liver sections stained with picrosirius red for collagen deposition and cytokeratin (CK) for neoplastic biliary cells, respectively. Stained slides were scanned using ChromaVision automated imaging scanning system (ChromaVision Medical Systems, San Juan Capistrano, CA), and quantified with MetaMorph software (Molecular Device, Sunnyvale, CA). Liver sections were scanned in a blinded manner, the captured bright-field images were digitized and the area and optical densities of the chromogen were quantified. To quantify the area of cholangiocarcinoma consisting of neoplastic biliary epithelium and fibrotic stromal matrix, the area of cholangiocarcinoma was measured manually with the MetaMorph program by tracing every tumor outline.

### Measurement of Liver Hydroxyproline Content

For the quantitation of total hepatic collagen, perfused livers were excised, lyophilized and weighed. Lyophilized tissue was then hydrolyzed in 2 N NaOH (final concentration: 100 µg dry tissue/µl) at 120°C for 25 minutes. Ehrlich’s reagent (Sigma, St. Louis, MO) was added to the hydrolysates for color reaction and absorbance was measured at 550 nm. Hepatic hydroxyproline content was expressed as ug hydroxyproline per mg of dry tissue. Trans-4-hydroxy-L-proline (Sigma) was used as a standard.

### RT-PCR Analysis of TGF-β gene Expression

Liver samples were placed in RNAlater (Ambion, Austin, TX) immediately after harvesting and stored at −80°C until analysis. Tissues were homogenized in 0.5 ml of Trizol reagent (Gibco, Grand Island, NY) and total RNA was extracted and purified using standard RNAeasy mini kit (Qiagen, Valencia CA). Specific fluorogenic probes were designed for rat TGF-β1, TGF-β2, and TGF-β3. Random hexamer primer was included in the reverse transcription polymerase chain reaction (RT-PCR); cDNA was generated from 2 µg of RNA by using BD Biosciences (Woburn, MA) Sprint PowerScript reagents according to manufacturer’s protocol. PCR amplification and analysis of PCR reaction were performed and monitored using an ABI Prism 7700 Sequence Detection System (TaqMan, Perkin-Elmer Applied Biosystem, Carlsbad, CA). For each cDNA sample the Ct value of each target sequence was normalized to ribosomal RNA-18S to obtain the relative levels of mRNA expression of TGF-β. PCR reactions were performed in a 96-well plate in the TaqMan system. Values are expressed as fold change vs. normal controls.

### Western Blotting

Liver tissues were homogenized in ice-cold Tris lysis buffer (BD Biosciences) containing 1% triton-X100 plus protease inhibitor cocktail tablet (Roche Diagnostics, Indianapolis, IN); protein concentration was measured by the BCA protein assay kit (Pierce, Rockford, IL). Samples were boiled for 10 min and equal amounts of protein were loaded onto NuPage 4–12% Bis-Tris gradient gel (Invitrogen, Carlsbad, CA). Proteins were transferred onto a nitrocellulose membrane (Amersham Biosciences, Piscataway, NJ). Blots were incubated in blocking buffer (1x TBS-0.1% Tween-20 with 5% (wt/vol) nonfat dry milk for 1 hour followed by incubation of the blots with a mouse anti-PAI-1 antibody (1∶1000, BD Sciences) at 4°C over night. Blots were then washed three times with washing buffer (1x TBS-0.1% Tween-20) and incubated with a secondary HRP-conjugated donkey anti-mouse IgG antibody (1∶5000, Amersham Biosciences) for 1 hour at room temperature. Bound antibody was visualized with the enhanced chemiluminescence reagent kit (Amersham Bioscience). Protein molecular weight standards (Invitrogen) were used to assess protein mass.

### Measurement of Total and Active PAI-1 Protein

Liver slices were homogenized at 4°C for 15 seconds at full speed on an Ultra-Turrax tissue homogenizer. Protein concentration of the homogenates was determined by BCA protein assay (Pierce). Total tissue type PAI-1 protein was determined using a solid phase enzyme immunoassay with two complementary murine monoclonal antibodies specific for PAI-1 (Hyphen BioMed, Neuville-sur-Oise, France). Briefly, tissue homogenates were incubated and bound to the first specific murine monoclonal pre-coated on solid phase through one epitope, recognized with the second specific antibody coupled to HRP. For active PAI-1 protein, tissue homogenates were incubated and bound to active recombinant tPA pre-coated in an ELISA plate. Only active PAI-1 reacts with tPA and is fixed on the solid phase. After washing, a mouse monoclonal antibody specific for human PAI-1 and coupled to HRP was applied and bound to active PAI-1. PAI-1 protein was measured at 450 nm, with a recombinant human PAI-1 used as a standard. Values were expressed as pg/mg protein.

### Immunostaining

Immunohistochemistry for cytokeratin (CK), a biliary epithelial marker [23], was carried out on deparaffinized liver sections. Briefly, sections were first treated at 85°C with an unmasking solution containing 1% of sodium citrate (Vector Laboratories, Burlingame, CA) and then incubated with a universal protein blocking reagent (DAKO, Carpinteria, CA) for 30 minutes at room temperature. Sections were then incubated for 1 hour with a rabbit polyclonal antibody against pan-CK (1∶100 dilution, DAKO). In our testing, this antibody was verified to recognize only biliary epithelial cells, without non-specific staining to other cell types or interstitial components. After washing, sections were incubated for 45 minutes with horseradish peroxidase (HRP) conjugated polymer anti-rabbit secondary antibody followed by incubation with DAB chromogen as a substrate (DAKO) for 1 minute. Sections were counter-stained with hematoxylin to visualize hepatic architecture.

### Statistical Analysis

Values are presented as mean ± SE. Statistical analysis of values was performed using One-Way ANOVA or the unpaired student *t* test, with *P* values <0.05 considered significant.

## Results

### Clinical Findings and Blood Chemistry Analysis

TAA dosing resulted in 13% mortality, which occurred primarily in the first 2 to 3 weeks of TAA administration. TAA dosing also caused a loss of body weight, which upon cessation of administration, was restored within 6 weeks to levels equivalent to normal controls regardless of PBS, 13C4 or 1D11 treatment (data not shown). Furthermore, during the time of TAA administration, there was corresponding evidence from the hepatic injury markers, ALT and AST, of cytotoxic insult, in agreement with published reports [24]. Upon cessation of TAA dosing, plasma liver enzymes over the course of 6 weeks returned to within normal levels (data not shown) and there were no significant differences among the PBS, 13C4 or 1D11 groups during this time (data not shown). Administration of 1D11 and 13C4 had no observable deleterious effect on the general health of animals during the period of investigation. No death of animals was found due solely to the administration of antibodies.

### Gross Appearance, Histopathological Assessment and Hydroxyproline Content

**Figure 2:**
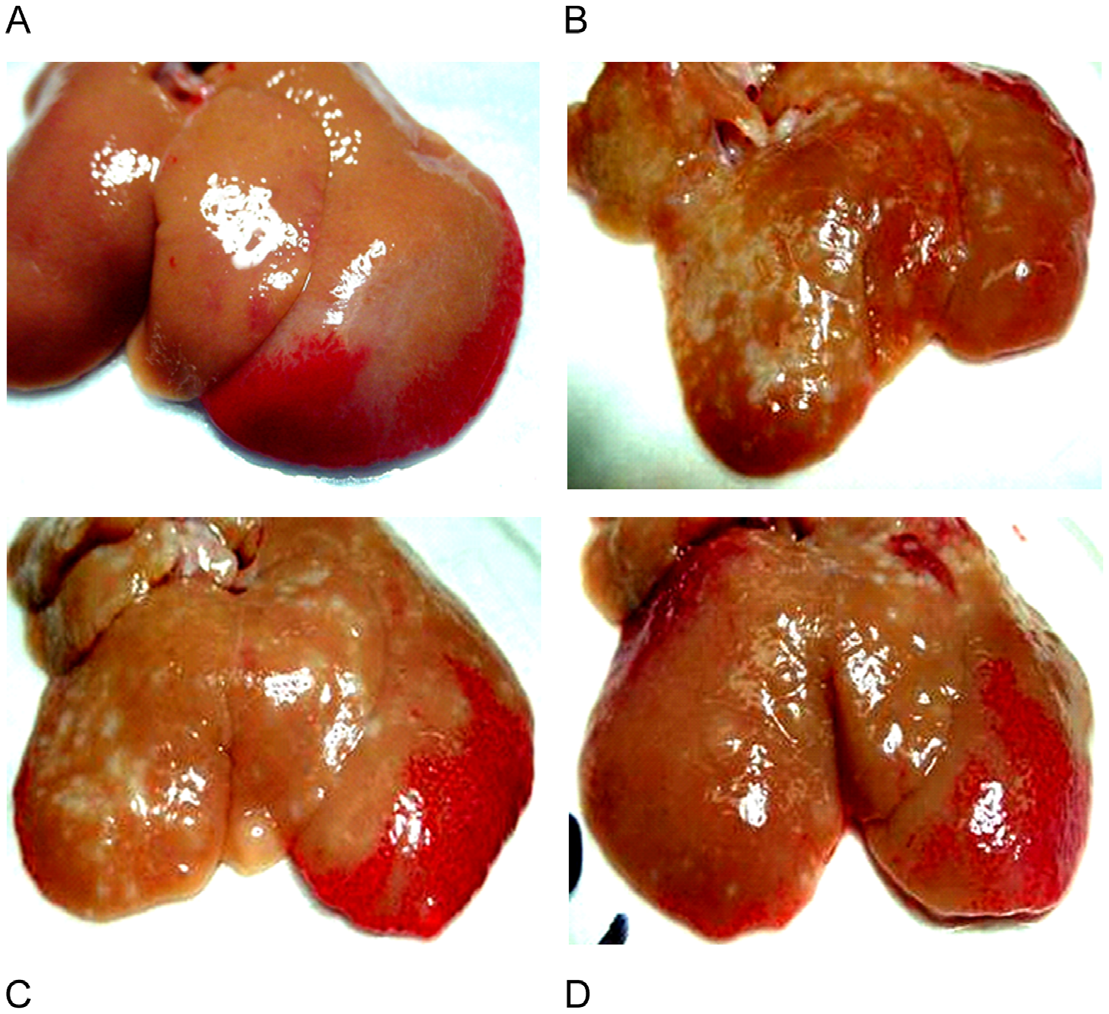
1D11 improved gross appearance of fibrotic livers. Representative photomicrographs at week 16 are shown. Gross examination of livers taken from normal controls (A), PBS untreated group (B), and control antibody group (C) showed stiff, swelling livers with rough, granular or nodular surface changes (yellowish in color) reminiscent of severe liver fibrosis in humans. Liver from rats that received 1D11 had much improved gross appearance (D), showing smoother surface and less nodular changes.

**Figure 3:**
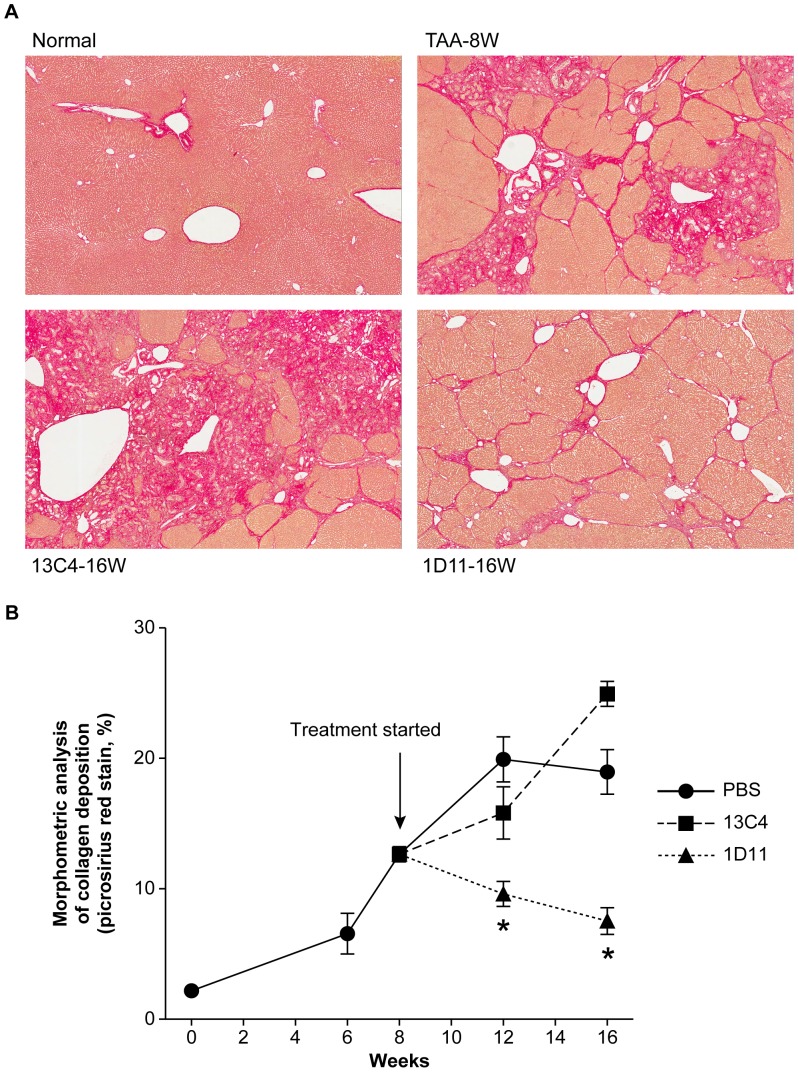
1D11 reversed pre-existing hepatic fibrosis. **A**. Representative photomicrographs of picrosirius red stained liver sections following treatment with TAA for 8 weeks (“baseline”; TAA-8W), or an additional 8 weeks with 13C4 (13C4–16W) or 1D11 (1D11–16W) after cessation of TAA administration. A normal rat liver is also shown (Normal). Livers of rats dosed with TAA for 8 weeks (TAA-8W) had substantial lesions with widely spread fibrous bands (septa), originating from portal areas and extending into the parenchyma. 8 weeks after stopping TAA dosing, fibrotic areas were further expanded, with extensive architectural disorganization and more fibrosis covering a greater percentage of the livers (13C4–16W). Treatment with 1D11 for 8 weeks significantly ameliorated the TAA-mediated histopathological lesions, as shown by an overall improvement in hepatic morphology (1D11–16W). Liver collagen deposition in rats treated with 1D11 was noticeably much reduced than the pre-established fibrosis seen at baseline (TAA-W8), shown in rats that were examined at baseline. Magnification: 40x. **B**. Morphometric analysis of picrosirius red stained sections. Significantly less collagen was deposited in livers treated with 1D11 (▴) for 4 weeks or 8 weeks, when compared to the PBS (•, * p<0.01) or 13C4 (▪, * p<0.01) treatment groups. Moreover, comparison of 1D11 treated livers to those at week 8 before therapeutic treatment started, showed significantly less collagen deposition (treatment for 4 weeks: 9.61%; 8 weeks: 7.53%, * p<0.05) than that harvested at baseline (week 8∶12.7%). Morphometric quantification further supports the histology of a reversal of pre-existing hepatic fibrosis when TGF-β was neutralized by 1D11. Values represent the percentage of collagen deposition in the total liver section and are expressed as mean ± SE (n = 8, except n = 4 for normal controls at week 0).

Gross examination showed stiff, swelling livers with rough, granular or nodular changes (yellowish in color) on the surface (Figure 2), resembling severe liver fibrosis in humans. Rats treated with 1D11 had much improved gross appearance (Figure 2D), showing smoother surface and less nodular change, as compared to rats dosed with PBS or 13C4 (Figure 2B and C). Histologic analysis showed widely spread fibrous bands (septa), originating from portal areas and extending into the hepatic parenchyma of rats upon cessation of TAA dosing (Figure 3A, TAA-8W). The disease groups, dosed with PBS (data not shown) or 13C4, showed further deterioration of hepatic architecture; portal fibrotic foci were more pronounced, with more apparent bridging fibrosis covering a greater percentage of the hepatic parenchyma. Treatment with 1D11 for 4 (data not shown) or 8 weeks significantly reduced TAA-induced collagen deposition/bridging fibrosis, and other lesions, as shown by an overall improvement in hepatic morphology (Figure 3A; 1D11-16w). The histologic appearance of samples from the 1D11 treated group also exhibited an improvement over baseline histology at the time point when TAA was stopped (Figure 3A; TAA-8w). These data strongly suggest that 1D11 was efficacious in treating pre-existing hepatic fibrosis and suggest some reversibility of histologically apparent fibrosis. Conventional blinded semi-quantitative scoring showed that rats dosed for 8 weeks with 1D11 had lower pathological scores (1.9±0.05), compared with those treated with PBS (3.4±0.1) or 13C4 (3.6±0.2). Morphometric analysis of liver sections (Figure 3B) revealed quantitative reduction in collagen deposition area (expressed as percentage of liver sections) in rats dosed with 1D11 for 4 (9.61%, p<0.05) or 8 weeks (7.53%, p<0.05) as compared to pre-existing fibrosis established before antibody treatment (12.65%). Progressive collagen deposition occurred in the control PBS and 13C4 groups. For comparison, the percent picrosirius red stain in normal liver was 2.14±0.5 at week 0 and 2.52±0.26 at week 16. The analysis of picrosirius red staining coupled with blinded histopathologic scoring suggests that therapeutic neutralization of TGF-β can block progressive hepatic fibrosis and may also reverse existing fibrosis. Hepatic hydroxyproline content was also measured in this study. It was significantly higher in rats treated with TAA followed with either PBS or 13C4 in comparison to normal controls (Table 1). TAA rats dosed with 1D11 had a significantly lower hydroxyproline content (1.04±0.09 ug/dry tissue) than either the 13C4 or PBS group (p<0.05). These data are consistent with and further support histological and morphometric analysis.

**Table 1:** 1D11 significantly reduces hepatic hydroxyproline content.

	Hydroxyproline content (ug/mg dry weight)
Normal controls	0. 50±0.02
PBS	1.54±0.16*
13C4	1.61±0.14*
1D11	1.04±0.09**

Animals were dosed with PBS, 13C4, and 1D11 for 8 weeks after cessation of TAA dosing.

* p<0.05 vs. normal controls and ** p<0.05 vs. PBS, 13C4 and normal control groups. Values represent mean ± SE.

### Effect of 1D11 on TGF-β1 Overexpression

**Figure 4:**
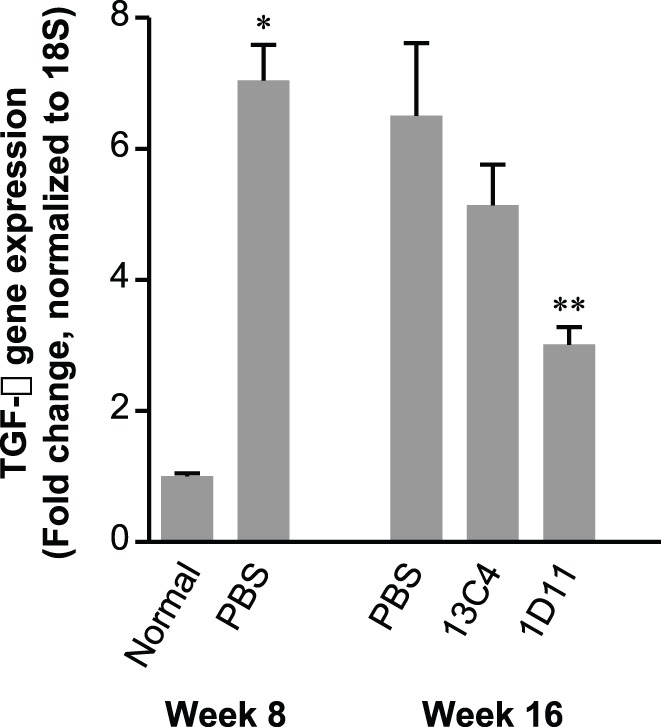
1D11decreased TGF-β1 mRNA. mRNA expression of TGF-β1 was analyzed by quantitative real-time RT-PCR for livers harvested at baseline (prior to treatment) and at week 16 (after treatment). TAA caused a sustained overexpression of TGF-β1 (>6 fold) throughout the study and treatment with 1D11 significantly reduced mRNA levels compared to baseline levels and in both PBS and 13C4 groups at end of study (week 16). Data at week 16 with 1D11 treatment suggest a reversal of fibrosis. Values are expressed as means ± SE, n = 8 per group; * p<0.01 vs. normal controls at week 8; ** p<0.05 vs. PBS or 13C4 group at week 16.

Hepatic expression of TGF-β1 was analyzed by real-time RT-PCR. At baseline (week 8) TGF-β1 expression was elevated 6 fold (Figure 4) with similar increases in the other two isoforms (data for TGF-β2 and β3 not shown). Interestingly, β3 rapidly returned to a level of expression slightly above normal upon cessation of TAA dosing, whereas TGF-β1 and β2 remained upregulated throughout the end of the study. TGF-β1 expression was significantly reduced with 1D11 dosing for 4 weeks (data not shown) or 8 weeks (p<0.05). Expression in control treatment groups were slightly, but not significantly reduced. These data suggest possible interruption of the known TGF-β autocrine regulation loop [25]. In contrast, TGF-β2 remained unchanged upon TGF-β neutralization (data not shown), suggesting that this isoform might not be accessible by 1D11, due to an intracellular localization in biliary epithelial cells (data not shown).

### Effect of 1D11 on Elevation of PAI-1

**Figure 5:**
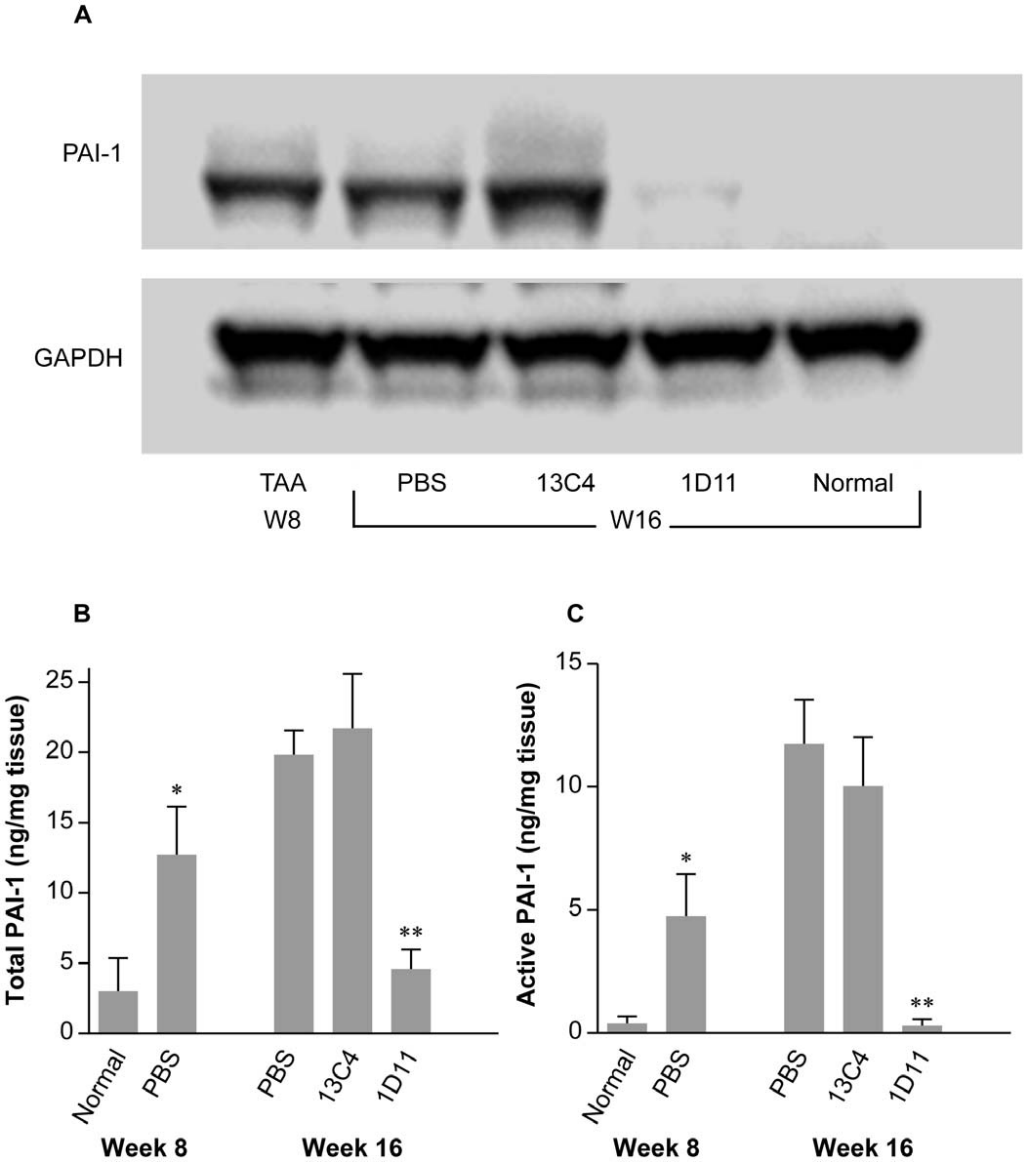
Increased PAI-1 protein in fibrotic liver was reduced in rats dosed with 1D11. **A**. A representative Western blot of pooled samples (n = 3) showed marked increase in PAI-1 expression before the start of therapeutic dosing at week 8. Levels were maintained in both PBS and 13C4 groups at the end of study at week 16. Levels of PAI-1 were normalized in livers treated with 1D11. **B and C**. Hepatic PAI-1 protein was also analyzed by ELISA. At the end of TAA administration, total (B) and active PAI-1 (C) were markedly elevated in fibrotic livers, with a further increase by week 16 in rats treated with PBS or 13C4. After 8 weeks of 1D11 dosing, total PAI-1 and active PAI-1 had returned to normal levels, confirming the immunoblotting data. Values are expressed as mean ± SE, n = 8; * p<0.01 vs. normal controls at week 8; ** p<0.01 vs. PBS or 13C4 group at week 16.

Elevation of PAI-1 may play a key role not only in fibrinolytic but also extracellular matrix turnover [9]–[10]. Because neutralization of TGF-β appeared to reverse existing hepatic fibrosis, we examined PAI-1 expression in this model. The expression of PAI-1 protein in livers was analyzed by Western blot, in which individual or pooled samples of liver homogenates from all treatment groups were assessed. A representative Western blot of pooled samples (n = 3) shows that PAI-1 protein was significantly increased at baseline and remained elevated in both control groups through the end of study (Figure 5A). In contrast, PAI-1 levels were nearly undetectable in rats dosed with 1D11. These findings are consistent with published data suggesting that PAI-1 over-expression is associated with suppressed ECM degradation in organ fibrosis [10]–[11], [25]. Further supporting this notion was the quantitative measurement of total PAI-1 (Figure 5B) and also active PAI-1 (Figure 5C) as determined in liver homogenates by ELISA. Interestingly, the increase in both total and active PAI-1 levels coincided with the progression of fibrosis, and was further elevated in both control groups at end of study. 1D11 dosing normalized both total PAI-1 and active PAI-1.

### Effects of 1D11 on TAA-induced CCA

**Figure 6:**
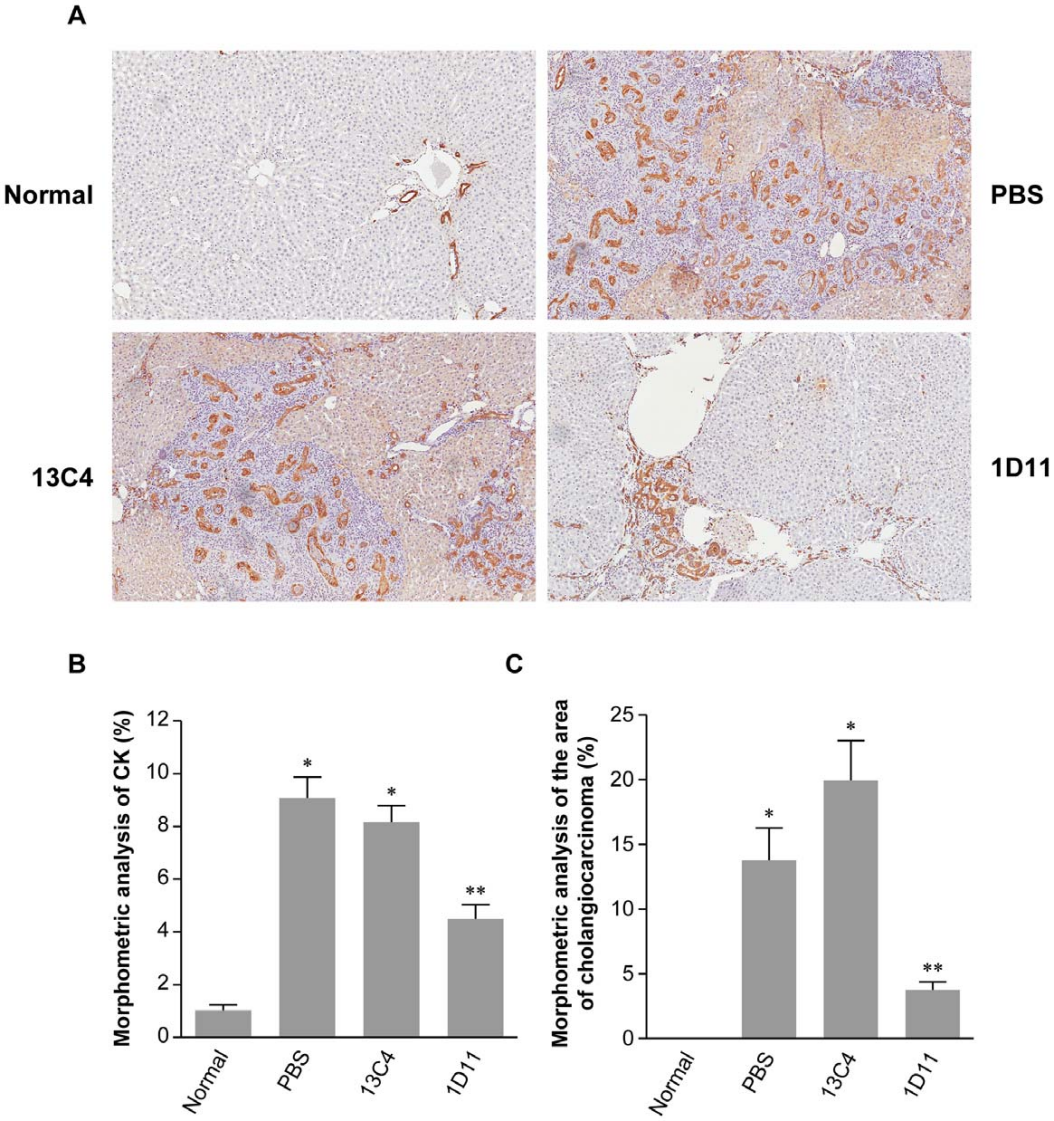
1D11 reduced cholangiocarcinomas induced by TAA. **A**. TAA induced profound neoplastic changes in biliary ductules (cholangiocarcinoma) at week 16. As visualized by CK staining, these neoplastic cells extended into the parenchyma, as fibrosis evolved (arrow heads). The neoplasia displayed a typical “intestinal metaplasia”-like appearance (arrows). The number of neoplastic biliary ductules or cells was much lower with 1D11 treatment, compared to the PBS or 13C4 groups. For comparison, a normal liver stained for CK is shown. Magnification: x100 **B**. CK staining was quantitated. The TAA-induced neoplasia was significantly reduced in rats treated with 1D11 for 8 weeks. In contrast, the neoplasia showed no reduction in rats dosed with PBS or 13C4. Values are expressed as mean ± SE of percentage of CK staining of the entire area of liver section. n = 8; * p<0.01 vs. normal controls; ** p<0.01 vs. PBS, 13C4 and normal control groups. **C**. To quantify the areas of cholangiocarcinoma, liver sections from the 16 time point were scanned by ChromaVision Imaging Analysis System. The area of the cholangiocarcinomas defined as neoplastic biliary epithelial cells plus stromal fibrotic tissue and infiltrated cells, was measured and expressed as a percentage of the total area of the liver section. Data revealed a striking reduction with 1D11 treatment, as compared to the PBS and 13C4 groups, reflecting a diminishment of cholangiocarcinoma by TGF-β neutralization. n = 8, * p<0.01 vs. normal controls; ** p<0.01 vs. PBS, 13C4 and normal control groups. Values represent mean ± SE.

TAA is also a potent carcinogen which induces CCA over a course of 10 to 22 weeks in rats [20]–[21]. In the present study, extensive microfoci of neoplastic biliary ductules embedded in fibrotic tissues were induced around week 8–12, and mass-forming CCA were seen at 16 weeks, which extended, as fibrosis evolved, into the parenchyma. The biliary neoplasia displayed a typical “intestinal metaplasia”-like appearance, with mucins found inside biliary epithelial cells or secreted into the lumen (Figure 6A). To better visualize and optimally quantify the area of these neoplastic biliary ductules, a monoclonal antibody against cytokeratin, an epithelial-specific marker, was applied using immunohistochemistry staining and qualified by morphometric analysis. All neoplastic biliary ductules, were immunostained, with no evidence of non-specific staining to other cell types or interstitial components. The number of ductules (or cells) was reduced substantially (45%) in rats dosed with 1D11 compared to the PBS or 13C4 control groups (Figure 6B) at study end. An average of 15 to 25 individual neoplastic foci were detected in rats from PBS or 13C4 control groups, whereas 3–7 foci were detected in 1D11 treated rats. To further quantify the area of CAA consisting largely of neoplastic biliary ductules and fibrotic stromal tissues, individual liver sections were qualified by morphometric analysis. This analysis (Figure 6C) showed a significant reduction in the area of CCA in rats treated with 1D11, as compared to the PBS or 13C4 treated control groups (p<0.01).

## Discussion

Numerous experimental and clinical studies have demonstrated a central role of TGF-β, and in particular β1, in liver fibrosis [7]–[9]. Studies using engineered TGF-β soluble receptors or siRNA interference technique have directly confirmed a causative role of this cytokine in liver fibrosis [16]–[19]. While the evidence linking TGF-β and fibrogenesis is substantial and raises possibilities for targeting this molecule as an appropriate therapeutic agent, it is necessary to demonstrate whether TGF-β antagonism can effectively treat pre-existing hepatic fibrosis. In the present study, we treated healthy rats with TAA for 8 weeks to establish hepatic fibrosis, which was histologically close to that seen in human liver cirrhosis. With fibrosis established, we examined the effect of administering a TGF-β neutralizing antibody, 1D11, on further fibrosis progression, and also whether there was evidence of regression from pre-existing fibrosis. After TAA withdrawal both control groups showed further deterioration of liver architecture mostly in the form of nodular damage and fibrous foci or septa bridging from portal area to portal area and to central areas. Also noted was substantial evolution of proliferating biliary epithelial neoplasia (CAA) within fibrous foci, consistent with earlier reports of TAA mediated hepatic lesions [20]–[21]. The degree of TAA-induced lesions correlated with the expression of TGF-β mRNA in the liver. TGF-β1, β2 and β3 were significantly upregulated after 8 weeks of TAA administration (6-fold increase for β1), and remained high in rats that received PBS or 13C4, except for TGF-β3, which declined but was still higher than normal controls upon the cessation of TAA administration.

Treatment with 1D11 reduced transcription of TGF-β1whereas the other two isoforms were unchanged, most likely due to their intracellular location in biliary epithelia. Reduced TGF-β1 expression and apparent interruption of the TGF-β1 autocine regulatory loop likely explain the therapeutic impact on pre-existing hepatic fibrosis. Histologically, a profound improvement in hepatic architecture with reduced number and area of fibrous foci or septa was seen in rats dosed with 1D11 for 8 weeks. Morphometric analysis, along with the measurement of hydroxyproline content, showed a significant decrease in collagen deposition in animals treated with 1D11. Of note, the two control groups showed progressive hepatic injury 4 and 8 weeks following cessation of TAA-induced injury, whereas the 1D11 treated group showed regression of fibrosis, significantly lower than levels recorded just before starting 1D11 treatment. Thus in this model there was no evidence of spontaneous resolution of injury, unlike other models [26] and, further, our data argue that antibody-mediated antagonism of TGF-β promotes regression of tissue fibrosis. We conclude that pathological hepatic fibrosis, at least in this preclinical model, is a dynamic situation and is potentially reversible. This may also be true in the human since recent studies examining hepatitis patients with long term antiviral therapy showed reduced liver cirrhosis [27]–[28]. Our results provided a platform for further mechanistic studies focusing on the reversibility of pathological fibrosis.

Accumulation of ECM proteins is caused not only by increased protein synthesis but also by decreased protein degradation. There is now a significant body of evidence that liver fibrosis is also a consequence of altered ECM degradation, in which PAI-1 may be implicated [10]–[11]. PAI-1 is the major physiologic inhibitor of tissue-type (t-PA) or urokinase-type plasminogen activator (µ-PA), both of which activate plasminogen to plasmin. Plasmin may also activate unrelated proteases to further promote resolution of fibrosis [29]. In the present study, a marked increase of PAI-1 protein occurred in parallel with fibrosis progression. PAI-1 remained elevated in rats treated with PBS or 13C4, but this was normalized in rats dosed with a TGF-β neutralizing antibody. Thus our data suggest that elevated PAI-1 contributed to the development of hepatic fibrosis through a TGF-β dependent manner. These data are consistent with several recent studies showing that; 1) direct manipulation of plasmin/PAI-1 with adenovirus-mediated transfer of siRNA decreased hepatic fibrosis in dimethylnitrosamine-induced and bile duct ligation-induced liver disease models [30]; 2) restoration of hepatic plasmin activity by a mutant, noninhibitory PAI-1 was also associated with decreased fibrotic matrix accumulation in this model [31] and, 3) higher levels of tPA activation have been associated with fewer hepatic lesions in PAI-1 (−/−) mice [32]. Reduced PAI-1 protein, along with attenuated TGF-β1 transcription may indicate an inhibition of TGF-β1/Smad signaling axis by 1D11, presumably with decreased phosphorylation of Smad2/3, a principal effector system of the signaling pathway contributing to fibrosis [25].

It is also known that TAA is a potent carcinogen and induces CCA in the rat, which recapitulates the histological features and progression of human CCA [20]–[21]. In the present study, TAA induced hepatic fibrosis, concomitant with development of neoplastic biliary epithelial ductules embedded in fibrotic/stromal tissues. The nature of this type of neoplastic biliary ductules was confirmed morphologically as a typical “intestinal metaplasia”-like appearance, identified by immunostaining of cytokeratins [23], and microscopic diagnosis by a board certified pathologist. The assessment of TAA-induced CCA by immunostaining also provided a way to determine the area of the neoplastic biliary ductules. Neoplastic bile ductules were remarkably fewer in number and in area in livers from rats dosed with 1D11. The diminishment of CCA occurred in parallel with an improved fibrosis index, suggesting a close relationship between these two. Indeed, elaboration of ECM proteins provides both a three-dimensional structure as well as matrix-cellular signals that promote tumorigenesis [16], [33]–[34]. With specific regard to CCA, Farazi PA et al. [35] have shown that increased production of type I and III collagens along with fibroblast recruitment stimulate biliary epithelium hyperplasia and subsequent progression to malignant intrahepatic tumors mice harboring a p53 mutant allele. Thus our findings are consistent with others’ observations suggesting that therapeutic reduction of hepatic fibrosis as a result of TGF-β neutralization represents a potential approach for the treatment of CCA.

To summarize, the present study demonstrates that 1D11, a murine monoclonal TGF-β neutralizing antibody, can reverse pre-existing clinically comparable hepatic fibrosis induced by an extended dosing of TAA. The regression of fibrosis was accompanied with a diminishment of cholangiocarcinoma, presumably linked to a reduction of fibrosis surrounding neoplastic ductules, associated with an inhibition of TGF-β and PAI-1. The data provide the first evidence that reversal of pre-existing hepatic fibrosis can be achieved upon TGF-β neutralization, providing proof-of-concept for considering TGF-β neutralizing antibody in the treatment of liver cirrhosis.
